# The Association between Stress Measured by Allostatic Load Score and Physiologic Dysregulation in African Immigrants: The Africans in America Study

**DOI:** 10.3389/fpubh.2016.00265

**Published:** 2016-11-25

**Authors:** Brianna A. Bingham, Michelle T. Duong, Madia Ricks, Lilian S. Mabundo, Rafeal L. Baker, Jean N. Utumatwishima, Margaret Udahogora, David Berrigan, Anne E. Sumner

**Affiliations:** ^1^Section on Ethnicity and Health, Diabetes, Endocrinology and Obesity Branch, National Institute of Diabetes, Digestive and Kidney Diseases, National Institutes of Health (NIH), Bethesda, MD, USA; ^2^National Institute of Minority Health and Health Disparities, National Institutes of Health (NIH), Bethesda, MD, USA; ^3^Dietetics Program, University of Maryland, College Park, MD, USA; ^4^Health Behavior Research Branch, Division of Cancer Control and Population Sciences, National Cancer Institute, National Institutes of Health (NIH), Bethesda, MD, USA

**Keywords:** allostatic load score, stress, health disparities, African immigrants, hypothalamic–pituitary–adrenal axis, sympathetic-adrenal-medullary axis

## Abstract

**Introduction:**

Allostatic load score (ALS) summarizes the physiological effect of stress on cardiovascular, metabolic and immune systems. As immigration is stressful, ALS could be affected.

**Objective:**

Associations between age of immigration, reason for immigration, and unhealthy assimilation behavior and ALS were determined in 238 African immigrants to the United States (age 40 ± 10, mean ± SD, range 21–64 years).

**Methods:**

ALS was calculated using 10 variables from three domains; cardiovascular (SBP, DBP, cholesterol, triglyceride, homocysteine), metabolic [BMI, A1C, albumin, estimated glomerular filtration rate (eGFR)], and immunological [high-sensitivity C-reactive protein (hsCRP)]. Variables were divided into sex-specific quartiles with high-risk defined by the highest quartile for each variable except for albumin and eGFR, which used the lowest quartile. One point was assigned if the variable was in the high-risk range and 0 if not. Unhealthy assimilation behavior was defined by a higher prevalence of smoking, alcohol consumption, or sedentary activity in immigrants who lived in the US for ≥10 years compare to <10 years.

**Results:**

Sixteen percent of the immigrants arrived in the US as children (age < 18 years); 84% arrived as adults (age ≥ 18 years). Compared to adulthood immigrants, childhood immigrants were younger (30 ± 7 vs. 42 ± 9, *P* < 0.01) but had lived in the US longer (20 ± 8 vs. 12 ± 9 years, *P* < 0.01). Age-adjusted ALS was similar in childhood and adulthood immigrants (2.78 ± 1.83 vs. 2.73 ± 1.69, *P* = 0.87). For adulthood immigrants, multiple regression analysis (adj *R*^2^ = 0.20) revealed older age at immigration and more years in the US were associated with higher ALS (both *P* < 0.05); whereas, current age, education, income, and gender had no significant influence (all *P* ≥ 0.4). The prevalence of smoking, alcohol intake, and physical activity did not differ in adulthood immigrants living in the US for ≥10 years vs. <10 years (all *P* ≥ 0.2). Reason for immigration was available for 77 participants. The reasons included: family reunification, lottery, marriage, work, education, and asylum. Compared to all other reasons combined, immigration for family reunification was associated with the lowest ALS (1.94 ± 1.51 vs. 3.03 ± 1.86, *P* = 0.03).

**Conclusion:**

African immigrants do not appear to respond to the stress of immigration by developing unhealthy assimilation behaviors. However, older age at immigration and increased duration of stay in the US are associated with higher ALS; whereas, family reunification is associated with lower ALS.

**Clinical Trials.gov Identifier:**

NCT00001853

## Introduction

Allostatic load is defined as stress-induced physiologic dysregulation caused by the chronic over-secretion of catecholamines and glucocorticoids (1, 2). Repeated exposure to these sympathetic-adrenal-medullary (SAM) and hypothalamic–pituitary–adrenal (HPA) hormones has adverse cardiovascular, metabolic, and immune consequences (1, 2). As allostatic load increases, so does the risk for chronic diseases, such as hypertension, obesity, cardiovascular disease, and diabetes (1, 2).

While allostatic load has been evaluated in African-Americans and to a lesser extent in foreign-born blacks, data specific to African immigrants are not available (3, 4). The reason for this lack of data is that health surveys either exclude African immigrants or place them in general categories, such as Black/African-American or foreign-born blacks (5, 6). Therefore, an exploration of how social and biological factors interact and influence the health of African immigrants has not previously been possible.

As African immigration to the United States has doubled every decade since 1970, in 2010, African immigrants represented 4% of the foreign-born adults in the United States (7). Importantly, three-quarters of the 1.6 million African immigrants living in the United States have arrived since 1990 (7). In short, African immigrants are a rapidly growing population who are historically and linguistically different from previous immigrants from Europe, South America, and the Caribbean. Therefore, research from these earlier immigrant populations, such as the development of unhealthy assimilation behavior as a way to cope with stress in the United States, must be re-assessed in African immigrants (8–11). The challenge is how to measure the biological consequences of chronic exposure to stress.

Even though the concept of measuring biologic stress by an allostatic load score (ALS) is decades old (1, 2), no consensus exists on which biomarkers to include in calculating ALS (12, 13). After reviewing the literature, we felt that studies of ALS, which focused on African-Americans and/or Hispanic immigrant populations, may have the most relevance to the African immigrant experience (12). Therefore, we chose the ALS formulation used by both Geronimus et al. and Kaestner et al. (4, 8). Working with data from NHANES 1999 to 2002, Geronimus et al. examined whether age, gender, or socioeconomic status could explain the adverse metabolic health experienced by African-Americans (4). With a focus on Mexican-born Americans, Kaestner et al. analyzed data from NHANES 1988 to 2004 to determine the effect of immigration on the development of unhealthy assimilation behaviors and metabolic markers of stress (8).

Our goal was to study the association between allostatic load and certain immigrant characteristics and health behaviors in African immigrants to the United States. For factors related to the immigrant experience, we examined: age of immigration, duration of stay in the United States, reason for immigration, and the prevalence of unhealthy behaviors specifically smoking, alcohol consumption, and sedentary lifestyle. For factors related to life in the United States, we examined education, income, and gender.

## Materials and Methods

The Africans in America cohort was designed to evaluate the cardiometabolic health status of African immigrants (9, 14, 15). Recruitment was achieved by newspaper advertisement (53%), previous participant referral (24%), flyers (7%), and the remainder from other sites, such as the NIH website and church gatherings. The NIDDK Institutional Review Board (http://ClinicalTrials.gov Identifier: NCT00001853) approved the study. Informed written consent was obtained prior to participation.

At the pre-enrollment telephone interview, all participants had to state that they were healthy, lived in the metropolitan Washington, DC, USA, area, were born in sub-Saharan Africa, and that both parents were black Africans born in sub-Saharan Africa. The African regions of origin of the participants were West (53%), Central (20%), and East (27%) (Table S1 in Supplementary Material). All enrollees were fluent in English, but 31% were born in Francophone countries (Table S1 in Supplementary Material).

The cohort had 309 enrollees, but the first 68 enrolled participants lacked data on high-sensitivity C-reactive protein (hsCRP). In addition, three immigrants had missing data. Therefore, 238 African immigrants (69% male, age 40 ± 10 (mean ± SD), range 21–64 years, 27.8 ± 4.4, range 18.2–41.2 kg/m^2^) were included in this evaluation.

Two outpatient visits were held at the NIH Clinical Research Center in Bethesda, MD, USA.

At Visit 1, a medical history, physical examination, and EKG were performed. Routine blood tests were done to document the absence of anemia as well as kidney, liver, and thyroid disease.

For Visit 2, participants fasted for 12 h and came to the Clinical Center at 7:00 a.m. The participant rested quietly for 20 min and then blood pressure (BP) and pulse were obtained three times approximately 5 min apart. The mean of the second and third readings were recorded. Glucose tolerance status was determined by a 2-h OGTT (Trutol 75; Custom Laboratories, Baltimore, MD, USA). In addition, fasting blood was drawn for lipids, hsCRP, homocysteine, and albumin levels.

### Diagnosis of Prediabetes and Diabetes

The diagnosis of prediabetes and diabetes was based on the American Diabetes Association criteria for glucose and A1C (16).

### Allostatic Load Equation

Allostatic load score was calculated based on 10 biomarkers from 3 categories: cardiovascular [systolic BP, diastolic BP, cholesterol, triglyceride (TG), homocysteine], metabolic [BMI, A1C, albumin, estimated glomerular filtration rate (eGFR)], and inflammatory hsCRP (4, 8). In the calculation of ALS, each variable was turned into a dichotomous variable with 1 point given if in the high-risk range and 0 if not. To determine high-risk, each biomarker was divided into sex-specific quartiles and then high risk was defined as a value above the 75th percentile for: systolic BP, diastolic BP, cholesterol, TG, homocysteine, hsCRP, A1C, BMI and below the 25th percentile for: albumin, eGFR (10). Individuals using antihypertensive medication were assigned to the high-risk category for BP. Allostatic score could range between 0 and 10. A higher ALS indicates worse stress associated physiologic dysfunction.

### Unhealthy Assimilation Behavior

Unhealthy assimilation behavior was evaluated in two ways. First, the prevalence of unhealthy assimilation behaviors was compared in adult and childhood immigrants.

Second, in adulthood immigrants, the prevalence of unhealthy assimilation behaviors was compared in those who had lived in the United States for ≥10 years to those who had lived in the United States for <10 years. 10 years of residence in the United States is the standard metric for the assessment of assimilation behaviors (8, 10).

To make these assessments of unhealthy assimilation, smoking, alcohol intake, and exercise were converted to dichotomous variables. Smoking was recorded as “no” if the immigrant either “never smoked” or “had no cigarette in 1 year.” Alcohol intake was reported as “no” if less than one alcoholic beverage was consumed per week (17). Exercise was coded as “no” if no vigorous or moderate activity was undertaken in the previous week (10).

### Analytic Measures

Cholesterol, TG, homocysteine, albumin, creatinine, and hsCRP were measured in plasma (Roche Cobas 6000 analyzer, Roche Diagnostics, Indianapolis, IN, USA). A1C values were determined using two different National Glycohemoglobin Standardization Program (NGSP)-certified instruments made by BioRad Laboratories (Hercules, CA, USA) using the same HPLC technology. A1C samples from the first 120 enrolled participants were measured on the Variant II instrument. The next 117 participants had A1C measurements performed on a D10 instrument. The correlation (*R*^2^) and mean bias between the Variant II and D10 instruments were 0.9934 and 0.07 (1.21%), respectively.

### Statistics

Unless otherwise stated, data are presented as mean ± SD. Group comparisons were made using unpaired *t*-tests, one-way analyses of variance, and the Chi-square tests. Pearson correlations were also calculated. Multiple regression analyses with ALS as the dependent variable and current age, age at immigration, years in the United States, gender, education, and income as independent variables were performed separately for adult and childhood immigrants. A *P*-values ≤0.05 were considered significant.

Analyses were performed with STATA14.0 (College Station, TX, USA).

## Results

The demographic and biological characteristics of the immigrants were analyzed in three different ways:

- Adulthood immigrants (arrived in the United States ≥18 years of age) vs. childhood immigrants (arrived in the United States <18 years of age) (Table 1);
- African region of origin (West vs. Central vs. East) (Table S2 in Supplementary Material); and
- Francophone vs. non-francophone countries (Table S3 in Supplementary Material).

**Table 1:** **Characteristics of adult and childhood immigrants**.

Parameter^a^ (mean ± SD)	Total cohort *n* = 238 (100%)	Adulthood immigrants^b^ *n* = 199 (84%)	Childhood immigrants^b^ *n* = 39 (16%)	*P*-value
Current age (years)	40 ± 10 (21–64)^c^	42 ± 9 (21–64)	30 ± 7 (22–50)	<0.01
Age at immigration (years)	27 ± 10 (0.5–61)	30 ± 8 (18–61)	11 ± 5 (0.5–17)	<0.01
Years in US (years)	13 ± 9 (0.1–40)	12 ± 9 (0.1–40)	20 ± 8 (5–36)	<0.01
Self-identify as African (*n* = 77) (%)^d^	87	85	93	0.41
Male (%)	69	72	51	0.01
Married (%)	49	53	28	<0.01
College graduate (%)	72	76	54	<0.01
Income (≥45k) (%)	53	51	64	0.14
Cigarette smoking (%)	5	6	3	0.44
Alcohol (≤1 drink/week) (%)	52	53	46	0.42
Sedentary (%)	35	36	28	0.34
Fasting glucose (mmol/L)	5.1 ± 0.7	5.1 ± 0.8	4.9 ± 0.5	0.08
2 h glucose (mmol/L)^e^	7.4 ± 2.3	7.6 ± 2.4	6.6 ± 1.9	0.02
Diabetes (%)	7	8	3	0.23
Systolic BP (mmHg)	121 ± 15	122 ± 15	116 ± 12	0.02
Diastolic BP (mmHg)	73 ± 10	73 ± 10	71 ± 10	0.12
Cholesterol (mmol/L)	4.3 ± 0.9	4.4 ± 0.9	4.1 ± 0.9	0.11
Triglyceride (mmol/L)	0.86 ± 0.42	0.89 ± 0.43	0.69 ± 0.33	<0.01
Homocysteine (μmol/L)	7.9 ± 2.6	8.0 ± 2.7	7.1 ± 1.9	0.05
BMI (kg/m^2^)	27.8 ± 4.4	28.2 ± 25.7	25.7 ± 3.7	<0.01
A1C (%)	5.5 ± 0.7	5.5 ± 0.7	5.4 ± 0.4	0.22
Albumin (mg/L)	40 ± 3	40 ± 3	39 ± 2	0.40
eGFR (mL/min/1.73 m^2^)^f^	108 ± 21	106 ± 20	116 ± 21	<0.01
hsCRP (nmol/L)	16.2 ± 22.3	17.3 ± 23.6	10.7 ± 13.4	0.09
Allostatic load score	2.74 ± 1.90	2.91 ± 1.94	1.90 ± 1.39	<0.01
Age-adjusted allostatic load score	2.74 ± 1.90	2.73 ± 1.69	2.78 ± 1.81	0.87

*^a^Data presented as mean ± SD*.

*^b^Adulthood immigrants came to the United States at age ≥18 years and childhood immigrants at age <18 years*.

*^c^Numbers in parentheses represent range*.

*^d^Data available in consecutively enrolled participants*.

*^e^Obtained during oral glucose tolerance test*.

*^f^Estimated glomerular filtration rate based on the Modification of Diet in Renal Disease (MDRD) Study Equation*.

### Adulthood Immigrants vs. Childhood Immigrants

All participants were adults at the time of enrollment (Table 1). However, the adulthood immigrants were older than the childhood immigrants (42 ± 9 vs. 30 ± 7 years, *P* < 0.01) but had lived in the United States for a shorter period of time than the childhood immigrants (12 ± 9 vs. 20 ± 8 years, *P* < 0.01). Both groups had a high rate of self-identification as Africans (85% vs. 93%, *P* = 0.41).

The majority of the adulthood immigrants were men (72%), but childhood immigrants were equally divided between men and women (51%).

For social variables, the adulthood immigrants were more likely to be married and have a college education than childhood immigrants (Table 1). However, income, frequency of smoking, alcohol intake, and sedentary lifestyle did not differ in adult and childhood immigrants.

Of the 10 factors that comprise the ALS, 4 were higher in adult than childhood immigrants (systolic BP, TG, homocysteine, and BMI), and 1 was lower (eGFR) (Table 1). Therefore, ALS was higher in the adulthood immigrants (2.91 ± 1.94 vs. 1.90 ± 1.59, *P* < 0.01). However, after adjusting for age, ALS did not differ between the adult and childhood immigrants (2.73 ± 1.69 vs. 2.78 ± 1.81, *P* = 0.87).

### African Region of Origin

As presented in Table S2 in Supplementary Material, East Africans were slightly younger than West Africans; whereas BMI was higher in Central than West and East Africans. In addition, East Africans had higher eGFR than the other two groups. However, ALS load score did not vary by African region of origin, and this was also true after adjusting ALS for age.

### Francophone vs. Non-Francophone Countries

After dividing the cohort into two groups according to whether the participants were from francophone or non-francophone countries, no difference was found in immigration, social, or metabolic variables (Table S3 in Supplementary Material). In addition, ALS was not different in the two groups.

### Demographic and Social Factors Associated with ALS

For the adulthood immigrants, Pearson correlations revealed that ALS was highly correlated with current age, age of immigration, and years in the United States (Table 2). In contrast, no significant correlations existed between ALS and either education or income (Table 2). With one exception, these findings were also found in the multiple regression analysis in which ALS was the dependent variable, and current age, age of immigration, years in the United States, education, and income were the independent variables (Table 3). In the multiple regression analysis, age of immigration and years in the United States remained highly significant but current age did not.

**Table 2:** **Pearson correlations with allostatic load score**.

Variable	Adulthood immigrants (*n* = 199)	Childhood immigrants (*n* = 39)
Current age	*r* = 0.45***	*r* = 0.47**
Age at immigration	*r* = 0.22**	*r* = 0.19
Years in the United States	*r* = 0.31***	*r* = 0.31
Education	*r* = −0.01	*r* = −0.19
Income	*r* = −0.01	*r* = 0.18

**P < 0.01.

***P < 0.001.

**Table 3:** **Multiple regression with ALS as dependent variable**.

(A) Adulthood immigrants (*n* = 199)			
*R*^2^ = 0.23, adj *R*^2^ = 0.20			
Independent variables	*β*-coefficient	SE	*P*-value
Current age	−0.064	0.073	0.39
Age at immigration	0.162	0.075	<0.05
Years in US	0.155	0.072	<0.05
Education	−0.070	0.102	0.49
Income	0.007	0.070	0.92
Gender	0.088	0.283	0.76

**(B) Childhood immigrants (*n* = 39)**			
***R*^2^ = 0.32, adj *R*^2^ = 0.19**			
**Independent variables**	***β*-coefficient**	**SE**	***P*-value**

Current age	−0.172	0.347	0.62
Age at immigration	0.263	0.351	0.46
Years in US	0.268	0.350	0.45
Education	−0.347	0.212	0.11
Income	0.128	0.135	0.35
Gender	0.127	0.449	0.78

For the childhood immigrants, current age was correlated with ALS. However, in the multiple regression, none of the five independent variables included (i.e., current age, age of immigration, years in the United States, education, and income) had a significant influence on ALS.

### Unhealthy Assimilation Behaviors

The three key behaviors associated with unhealthy assimilation were compared in adulthood immigrants who had lived in the United States for less than 10 years vs. 10 years or more. While the adulthood immigrants who had been in the United States for 10 years or more were older and had higher ALS than their counterparts who had lived in the United States for <10 years, there was no difference in the prevalence of smoking, alcohol intake, or sedentary behavior (Table 4).

**Table 4:** **Comparison of assimilation behaviors in adulthood immigration according to duration of stay in the United States**.

Variable	<10 years *n* = 95	≥10 years *n* = 104	*P*-value
Age (years)	36 ± 8	46 ± 8	<0.01
Allostatic load score	2.45 ± 1.95	3.32 ± 1.85	<0.01
Cigarette smoking (%)	5	6	0.88
No alcohol intake (%)	52	55	0.65
% Sedentary	41	32	0.17

In addition, unhealthy assimilation behaviors were compared in all adulthood vs. childhood immigrants, and no significant difference was detected (all *P* > 0.3) (Table 1).

### Reason for Immigration

**Figure 1:**
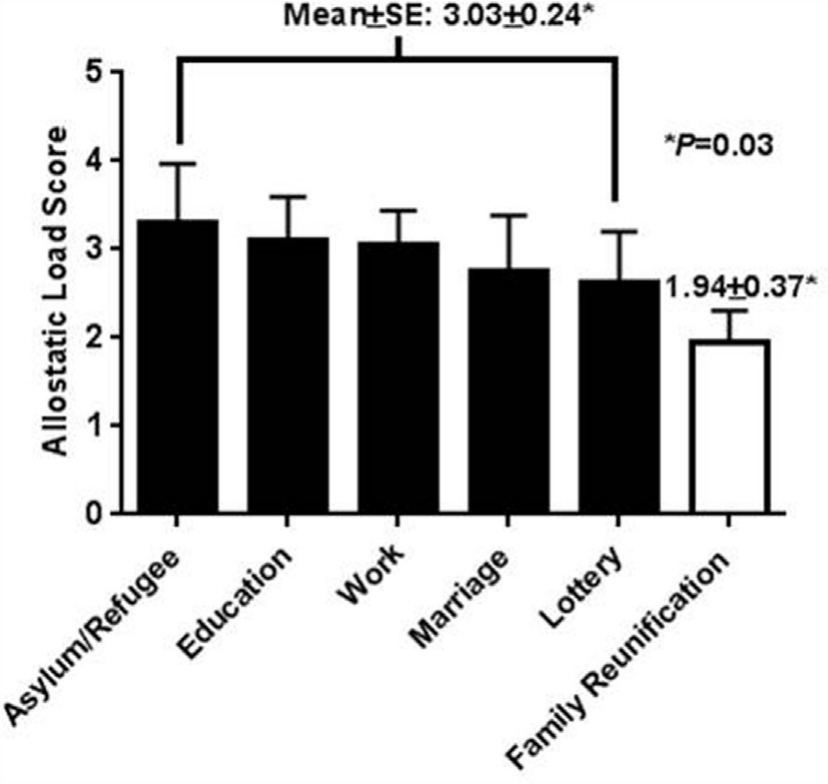
**Allostatic load score according to reason for immigration**. Data presented as mean ± SE. Difference in allostatic load score between all causes combined vs. family reunification is *P* = 0.03.

The reasons for immigration from the highest to the lowest ALS were: asylum/refugee (3.30 ± 2.11), education (3.10 ± 2.22), work (3.06 ± 1.63), marriage (2.75 ± 1.26), lottery (2.63 ± 1.60), and family reunification (1.94 ± 1.52) (Figure 1). When ALS for family reunification was compared to all other causes for immigration, ALS was significantly lower for family reunification (1.94 ± 1.51 vs. 3.03 ± 1.86, *P* = 0.03).

## Discussion

Evaluating physiologic stress in African immigrants by ALS, our main findings were (1) family reunification was associated with the lowest ALS; (2) unhealthy assimilation behaviors were not characteristic of African immigrants; (3) older age of immigration and increased duration of stay in the United States were associated with more stress and higher ALS in adulthood immigrants; and (4) higher education, greater income, and gender in African immigrants did not appear to influence ALS.

### Reason for Immigration

We considered whether the degree of stress triggered by immigration was modified by the reason for immigration. Yet, we could not find any publications in either foreign-born blacks or Mexican-born Americans, which relate ALS to reason for immigration. In our study, the reason for immigration was available in the 77 most recently enrolled participants. From highest to lowest ALS, the reasons for immigration were: asylum/refugee, education, work, marriage, lottery, and family reunification (Figure 1). Marriage was viewed as a category to separate from family reunification, because the married state is stressful and represents starting a new family rather than uniting an existing family (3). ALS was significantly lower for family reunification vs. all other causes combined (*P* = 0.03) (Figure 1). This differential in ALS across reasons for immigration suggests that family reunification is highly beneficial and presumably associated with lower levels of stress hormones and less metabolic dysregulation.

### Unhealthy Assimilation Behaviors

Unhealthy assimilation is based on the concept that after living in the United States, immigrants acquire unhealthy lifestyle practices, such as cigarette smoking, alcohol use, and a sedentary lifestyle (3, 8, 10, 11). Overall, unhealthy assimilation behaviors are considered to be a reaction to stress (1, 8). However, unhealthy assimilation behaviors also lead to changes in the parameters used to calculate the ALS (1). Therefore, a self-perpetuating relationship is believed to exist between unhealthy assimilation behavior and ALS (1). But whether unhealthy assimilation behaviors occur in African immigrants has not previously been investigated.

We did not detect any difference in the prevalence of unhealthy assimilation behaviors in adulthood immigrants who had been in the United States ≥10 years compared to <10 years (Table 4). In addition, the rates of these behaviors were as low in childhood immigrants as they were in adulthood immigrants (Table 1). Therefore, independent of duration of stay in the United States or age of immigration, unhealthy assimilation behaviors are not characteristic of the African immigrant experience.

The development of unhealthy assimilation behaviors in Mexican-born Americans has been attributed to acculturation and decreased adherence to birth country traditions (8). In this regard, Mexican and African immigrants may be different because of the African immigrants queried on this, 85% of the adulthood immigrants and 93% of the childhood immigrants self-identified as African. This high rate of self-identification as African may contribute to why African immigrants, as a group, do not appear to acquire unhealthy assimilation behaviors.

### Immigration-Specific Factors: Age and Age of Immigration

There is a general consensus that ALS increases with age (3, 4). Indeed, we found that age was positively correlated with ALS in both adulthood and childhood immigrants (Table 2). Importantly, childhood immigrants were younger and had lower ALS than adulthood immigrants, but after adjusting for age, ALS did not differ in adult and childhood immigrants (Table 1).

Beyond current age, we examined the association of age of immigration with ALS. With both current age and age of immigration entered into the multiple regression analysis as independent variables, age of entry rather than overall age was a key determinant of ALS in adulthood immigrants (Table 3A). This suggests that immigration at the age of 60 years is more stressful than being 60 years old and having immigrated at the age of 40 years.

The observation that the age of immigration influences ALS was robust in adulthood but not childhood immigrants. In childhood immigrants no influence of the age of immigration on ALS was detectable by either Pearson correlation (Table 2) or multiple regression (Table 3B). However, as the age range of immigration for the childhood immigrants was relatively narrow (6 months to 17 years) compared to the >40-year span (18–61 years) for the adulthood immigrants, correlations may be more difficult to detect in the childhood immigrants.

### Duration of Time Spent in the United States

For both foreign-born blacks and older Mexican-born Americans, ALS increases as duration of time in the United States increases (3, 8, 10). According to both Pearson correlation and multiple regression analysis, this appears to be true in adulthood African immigrants as well. Doamekpor and Dinwiddie have proposed that a major reason why foreign-born blacks have an increase in ALS with longer duration of stay in the United States is that transitioning from life in a black majority country to a country in which they are a minority population is very stressful (3). For African immigrants, we add the hypotheses that chronic concern about family members not living in the United States as well as ongoing worry about conditions in their homeland and the realization that the transition to the United States may be permanent.

The relationship of duration of stay in the United States to ALS was different in childhood immigrants than adulthood immigrants. By both Pearson correlation and multiple regression analyses, duration of stay in the United States did not influence ALS in childhood immigrants (Tables 2 and 3B). Similarly, Kaestner et al. reported that even though younger Mexican immigrants (<45 years of age) had been in the United States longer than older Mexican immigrants (≥45 years), ALS was not influenced in the younger Mexican immigrants by duration of stay in the United States (8). Kaestner et al. attributed the lack of effect of duration of stay on ALS in younger Mexican immigrants to the resilience of youth (8).

### Education and Income

For African-Americans, higher socioeconomic status is not associated with a decrease in stress or ALS (4). The failure of income and education to alleviate stress and ALS in African-Americans is attributed to high-effort coping, racism, and unconscious bias (4). It has been theorized that because African immigrants have not experienced the legacy of slavery or Jim Crow laws or other more current aspects of American racism, they are relatively immune to the psychological stress of discrimination. Alternatively, it has been suggested that adjusting to life in a country where blacks are a minority rather than a majority is very stressful for African immigrants (3).

We found that similar to African-Americans (4) neither higher education nor higher income was associated with lower ALS in African immigrants (Tables 2 and 3A,B). While racism or perceived discrimination within the context of life in America may be contributing, there may be reasons unique to African immigrants as to why neither education nor income mitigate stress and lower ALS. First, education may not match employment. Graduate degrees from universities in Africa may not be recognized in the United States, and highly educated immigrants may be working in low paying jobs. Second, if African immigrants living in the United States are sending money to relatives in Africa, income may not match financial security.

### Gender

Throughout the lifespan ALS is higher in African-American women than men (4). This gender difference has been attributed to the fact that African-American women experience the double jeopardy of sexism and racism (4). However, in African immigrants, we found no gender difference in ALS. The fact that ALS differs in African-American men and women but not in African immigrant men and women may be secondary to cultural differences in the role of women.

We note with interest that the adulthood immigrants were 72% male but the childhood immigrants were 51% male (*P* = 0.01). The imbalance between male and female participants among the adulthood immigrants is consistent with the report of the Migration Policy Institute, which states that among adult Africans, more men immigrate than women (http://www.migrationpolicy.org/article/african-immigrants-united-states). When families come to the US, the distribution of male and female children would be expected to be similar.

### Limitations

The limitations of this study include the cross-sectional design, the use of a convenience sample, the fact that biologic parameters were measured only once, the relatively small number of childhood immigrants, the disproportionately low number of women, the lack of information on illicit drug use as well as the limited information on exercise. In the absence of centrally collected statistics on African immigrant health, it is not possible to determine the degree to which the Africans in America cohort are representative of African immigrants in the United States. However, there are two reasons to suggest that our data may be representative. First, the United States Census Bureau has reported that the majority of Africa immigrants living in the United States are from West African countries. We found that 53% of the enrollees in our study were from West African countries compared to 20% and 27% from Central and East African countries, respectively (Table S1 in Supplementary Material). Second, the 7% prevalence of diabetes in our African immigrant cohort was remarkably similar to the 8% prevalence reported by Canadian governmental agencies in African immigrants to Canada (18). Furthermore, in a population based study in Nigeria, oral glucose tolerance tests were performed and the prevalence of undiagnosed diabetes was 7% (19).

## Conclusion

Stress leads to the secretion of hormones, which promote physiologic dysregulation. Using ALS as a measure of stress-induced physiologic dysregulation, our study suggests that the stress of living in America may be minimized by reuniting families. However, in adulthood immigrants, older age of immigration, and increased duration of stay increases stress. But this stress does not appear to lead in African immigrants to the development of unhealthy assimilation behaviors. Similar to African-Americans (4), income and education may not lower ALS in Africans immigrants. Reasons for the inability to detect a decrease in ALS with higher education and income may overlap with the African-American experience, but additional immigration-specific causes need consideration. If immigrants are sending money to relatives living outside the United States, income may not match financial security. As high ALS may precipitate or exacerbate diseases in the cardiovascular, metabolic, and immune realm, promoting better health for African immigrants living in the United States requires an understanding of the stress they experience.

## Author Contributions

All the authors (BB, MD, MR, LM, RB, JU, MU, DB, and AS) declare that they: (a) have made substantial contribution to the conception and design of this work and participated in the acquisition, analyses, or interpretation of the data; (b) drafted the manuscript or provided critically important intellectual content which led to revisions; (c) gave final approval of the version submitted for publication; and (d) agreed to be accountable for all aspects of the work to ensure that questions related to accuracy and integrity of any part of the work are appropriately investigated and resolved.

## Conflict of Interest Statement

The authors declare that they have no financial or commercial interests or relationships that would perceived as a conflict of interest or a potential conflict of interest.
