# Supplementary material for: The Association between Stress Measured by Allostatic Load Score and Physiologic Dysregulation in African Immigrants: The Africans in America Study
Source: Front Public Health. 2016 Nov 25;4:265. doi: 10.3389/fpubh.2016.00265 (PMC5122568; doi:10.3389/fpubh.2016.00265)
Supplement: Supplementary file 1 [file table_1.docx]

**Supplement Table 1: Distribution of Participants from Countries in Africa according to Francophone Status**

|  | **West 53% (n=127)** | **Central** **20% (n=48)** | **East 27% (n=63)** |
| --- | --- | --- | --- |
| **Francophone** | Other^1,2^: 3% (n=9)  Ivory Coast: 2% (n=6)  Togo: 2% (n=5) | Cameroon: 17% (n=40)  Other ^3^: 3% (n=8) | Other ^4^: 3% (n=6) |
| **Non Francophone** | Nigeria: 19% (n=44)  Ghana: 18% (n=43)  Sierra Leone: 5% (n=11)  Gambia: 2% (n=4)  Liberia: 2% (n=5) |  | Ethiopia: 10% (n=24)  Kenya: 7% (n=17)  Uganda: 4% (n=9)  Other ^5^: 3% (n=7) |

^1^Other refers to countries with 3 participants or less. ^2^Benin, Burkina Faso, Guinea, Mali and Senegal ^3^Democratic Republic of Congo, Republic of Congo and Central African Republic ^4^Burundi and Rwanda ^5^Eritrea, Somalia, Sudan and Tanzania

**Supplement Table 2: Characteristics of African Immigrants According to Region of Origin**

| **Parameter**  **(mean±SD)** | **West**  **n=127**  **(53%)** | **Central**  **n=48**  **(20%)** | **East**  **n=63**  **(27%)** | ***P*-value** |
| --- | --- | --- | --- | --- |
| **Current Age (y)** | 41±11 | 40±11 | 37±8 | 0.04^1^ |
| **Age at Immigration (y)** | 27±11 | 28±9 | 24±10 | 0.11 |
| **Years in US (y)** | 14±10 | 12±9 | 13±8 | 0.68 |
| **Self-Identify as African (n=77)** | 89% | 91% | 79% | 0.50 |
|  |  |  |  |  |
| **Male (%)** | 69% | 73% | 64% | 0.55 |
|  |  |  |  |  |
| **Married (%)** | 45% | 56% | 51% | 0.38 |
| **College Graduate (%)** | 72% | 75% | 70% | 0.83 |
| **Income (≥45k)** | 50% | 54% | 60% | 0.38 |
| **Cigarette Smoking (%)** | 6% | 2% | 6% | 0.56 |
| **Alcohol (≤1 drink/week)** | 40% | 67% | 49% | <0.01 |
| **Sedentary (%)** | 41% | 23% | 32% | 0.07 |
|  |  |  |  |  |
| **Fasting glucose (mmol/L)** | 5.1±0.9 | 5.1±0.6 | 5.1±0.5 | 0.98 |
| **2h glucose (mmol/L)** | 7.4±2.6 | 7.7±1.9 | 7.3±2.2 | 0.64 |
| **Diabetes (%)** | 6% | 8% | 8% | 0.86 |
|  |  |  |  |  |
| 1. **Systolic BP (mmHg)** | 122±15 | 123 ± 13 | 117±15 | 0.06 |
| 1. **Diastolic BP (mmHg)** | 73±10 | 74±11 | 72±10 | 0.49 |
| 1. **Cholesterol (mmol/L)** | 4.5±0.9 | 4.2±0.9 | 4.2±0.9 | 0.06 |
| 1. **Triglyceride (mmol/L)** | 0.88±0.45 | 0.78±0.36 | 0.86±0.40 | 0.30 |
| 1. **Homocysteine (µmol/L)** | 7.9±3.2 | 8.1±2.0 | 7.7±2.1 | 0.71 |
| 1. **BMI (kg/m^2^)** | 27.6±4.4 | 29.2±5.0 | 27.2±3.8 | 0.06 |
| 1. **A1C (%)** | 5.5±0.8 | 5.5±0.5 | 5.4±0.4 | 0.26 |
| 1. **Albumin (mg/L)** | 40±3 | 39±3 | 39±2 | 0.18 |
| 1. **eGFR (mL/min/1.73m^2^)^3^** | 102±18 | 107±20 | 121±21 | <0.01^2^ |
| 1. **hsCRP (nmol/L)** | 15.0±22.1 | 17.8±22.8 | 17.4±22.6 | 0.67 |
|  |  |  |  |  |
| **Allostatic Load Score** | 2.93±2.01 | 2.81±2.01 | 2.30±1.49 | 0.10 |
| **Age-adjusted Allostatic Load Score** | 2.82±1.65 | 2.77±1.68 | 2.55±1.66 | 0.29 |

^1^Difference between West and East Africans significant at P=0.04 ^2^Difference between both East and West Africans and East and Central Africans significant at P<0.01 ^3^estimated Glomerular Filtration Rate based on the Modification of Diet in Renal Disease (MDRD) Study Equation

**Supplement Table 3: Characteristics of Immigrants According to Francophone Status of Country of Origin**

| **Parameter**  **(mean±SD)** | **Francophone**  **n=74**  **(31%)** | **NonFrancophone**  **n=164**  **(69%)** | ***P*-value** |
| --- | --- | --- | --- |
| **Current Age (y)** | 40±10 | 40±10 | 0.99 |
| **Age at Immigration (y)** | 42±9 | 30±7 | <0.01 |
| **Years in United States (y)** | 12±9 | 20±8 | <0.01 |
| **Self-Identify: African (n=76)** | 82% | 88% | 0.53 |
|  |  |  |  |
| **Male (%)** | 70% | 68% | 0.69 |
|  |  |  |  |
| **Married (%)** | 54% | 46% | 0.27 |
| **College Graduate (%)** | 72% | 73% | 0.88 |
| **Median Income (≥45k)** | 50% | 55% | 0.49 |
| **Cigarette Smoking (%)** | 5% | 5% | 0.86 |
| **Alcohol (≤1 drink/week)** | 44% | 56% | 0.07 |
| **Sedentary (%)** | 65% | 65% | 0.96 |
|  |  |  |  |
| **Fasting glucose (mmol/L)** | 5.0±0.6 | 5.2±0.8 | 0.11 |
| **2h glucose (mmol/L)** | 7.3±1.9 | 7.4±2.6 | 0.56 |
| **Diabetes (%)** | 5% | 8% | 0.48 |
|  |  |  |  |
| 1. **Systolic BP (mmHg)** | 122±14 | 121±15 | 0.45 |
| 1. **Diastolic BP (mmHg)** | 73±11 | 73±10 | 0.98 |
| 1. **Cholesterol (mmol/L)** | 4.4±1.0 | 4.3±0.9 | 0.87 |
| 1. **Triglyceride (mmol/L)** | 0.80±0.36 | 0.88±0.44 | 0.20 |
| 1. **Homocysteine (µmol/L)** | 7.7±2.1 | 7.9±2.8 | 0.57 |
| 1. **BMI (kg/m^2^)** | 28.2±4.9 | 27.6±4.2 | 0.31 |
| 1. **A1C (%)** | 5.5±0.5 | 5.5±0.7 | 0.70 |
| 1. **Albumin (mg/L)** | 39±3 | 40±3 | 0.13 |
| 1. **eGFR (mL/min/1.73m^2^)^1^** | 109±19 | 108±21 | 0.73 |
| 1. **hsCRP (nmol/L)** | 17.9±26.2 | 15.4±20.4 | 0.42 |
|  |  |  |  |
| **Allostatic Load Score** | 2.73±1.99 | 2.74±1.86 | 0.96 |

^1^estimated Glomerular Filtration Rate based on the Modification of Diet in Renal Disease (MDRD) Study Equation
